# Diaqua­bis­(5-carb­oxy-2-ethyl-1*H*-imidazole-4-carboxyl­ato-κ^2^ *N* ^3^,*O* ^4^)cobalt(II) trihydrate

**DOI:** 10.1107/S1600536812008902

**Published:** 2012-03-03

**Authors:** Dong-Liang Miao, Shi-Jie Li, Wen-Dong Song, Shao-Wei Tong, Seik Weng Ng

**Affiliations:** a College of Food Science and Technology, Guangdong Ocean University, Zhanjiang 524088, People’s Republic of China; b School of Environment Science and Engineering, Donghua University, Shanghai 200051, People’s Republic of China; c College of Science, Guangdong Ocean University, Zhanjiang 524088, People’s Republic of China; d Department of Chemistry, University of Malaya, Kuala Lumpur 50603, Malaysia; e Chemistry Department, Faculty of Science, King Abdulaziz University, PO Box 80203 Jeddah, Saudi Arabia

## Abstract

In the title compound, [Co(C_7_H_7_N_2_O_4_)_2_(H_2_O)_2_]·3H_2_O, the Co^II^ cation, located on an inversion center, is *N*,*O*-chelated by two 5-carboxy-2-ethyl-1*H*-imidazole-4-carboxylate anions and further coordinated by two water mol­ecules in a distorted octa­hedral geometry. Only one carboxy group of the anion is deprotonated, and the two carboxyl groups of the same anion are linked *via* an intra­molecular O—H⋯O hydrogen bond. One of the lattice water mol­ecules is located on an inversion center, its H atom equally disordered over two positions. One of H atoms of another lattice water mol­ecules is also equally disordered over two sites. Water H atoms and the amino H atom all are involved in an inter­molecular hydrogen-bonded network in the crystal.

## Related literature

For related metal complexes with imidazole-4,5-dicarboxyl­ate ligands, see: Fan *et al.* (2010 ▶); Li *et al.* (2011 ▶); Yan *et al.* (2010 ▶); Song *et al.* (2010 ▶); He *et al.* (2010 ▶).
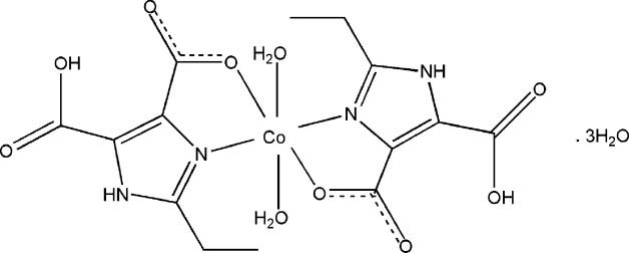


## Experimental

### 

#### Crystal data

[Co(C_7_H_7_N_2_O_4_)_2_(H_2_O)_2_]·3H_2_O
*M* *_r_* = 515.30
Triclinic, 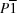

*a* = 7.1615 (14) Å
*b* = 8.8729 (18) Å
*c* = 9.3815 (19) Å
α = 66.06 (3)°
β = 88.66 (3)°
γ = 70.97 (3)°
*V* = 511.0 (3) Å^3^
*Z* = 1
Mo *K*α radiation
μ = 0.92 mm^−1^
*T* = 293 K
0.20 × 0.18 × 0.15 mm

#### Data collection

Bruker SMART APEXII diffractometer
Absorption correction: multi-scan (*SADABS*; Bruker, 2001 ▶) *T*_min_ = 0.781, *T*_max_ = 0.781
5086 measured reflections
2319 independent reflections
1578 reflections with *I* > 2σ(*I*)
*R*_int_ = 0.029

#### Refinement

*R*[*F* ^2^ > 2σ(*F* ^2^)] = 0.035
*wR*(*F* ^2^) = 0.102
*S* = 1.01
2319 reflections
149 parameters
5 restraints
H-atom parameters constrained
Δρ_max_ = 0.59 e Å^−3^
Δρ_min_ = −0.73 e Å^−3^

### 

Data collection: *APEX2* (Bruker, 2007 ▶); cell refinement: *SAINT* (Bruker, 2007 ▶); data reduction: *SAINT*; program(s) used to solve structure: *SHELXTL* (Sheldrick, 2008 ▶); program(s) used to refine structure: *SHELXTL*; molecular graphics: *SHELXTL*; software used to prepare material for publication: *SHELXTL*.

## Supplementary Material

Crystal structure: contains datablock(s) I, global. DOI: 10.1107/S1600536812008902/xu5468sup1.cif

Structure factors: contains datablock(s) I. DOI: 10.1107/S1600536812008902/xu5468Isup2.hkl

Additional supplementary materials:  crystallographic information; 3D view; checkCIF report

## Figures and Tables

**Table 1:** Selected bond lengths (Å)

Co1—O1	2.153 (2)
Co1—O1*W*	2.064 (2)
Co1—N2	2.123 (2)

**Table 2:** Hydrogen-bond geometry (Å, °)

*D*—H⋯*A*	*D*—H	H⋯*A*	*D*⋯*A*	*D*—H⋯*A*
N1—H1⋯O2*W*^i^	0.86	1.96	2.786 (4)	160
O3—H3⋯O2	0.85	1.63	2.471 (3)	171
O1*W*—H1*W*⋯O4^ii^	0.85	1.86	2.708 (3)	173
O1*W*—H2*W*⋯O3^iii^	0.85	1.94	2.763 (3)	161
O2*W*—H3*W*⋯O1	0.85	2.33	3.077 (4)	147
O2*W*—H3*W*⋯O3*W*	0.85	2.44	3.091 (3)	134
O2*W*—H4*W*⋯O2*W*^iv^	0.85	2.04	2.883 (6)	172
O2*W*—H7*W*⋯O4^v^	0.85	2.35	3.120 (4)	151
O3*W*—H5*W*⋯O2^vi^	0.85	2.37	3.040 (2)	136
O3*W*—H6*W*⋯O1	0.85	2.26	3.031 (2)	151
O3*W*—H6*W*⋯O2	0.85	2.43	3.040 (2)	129

Symmetry codes: (i) 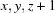; (ii) 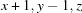; (iii) 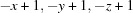; (iv) 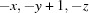; (v) 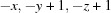; (vi) 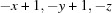.

**Fig. 1.**
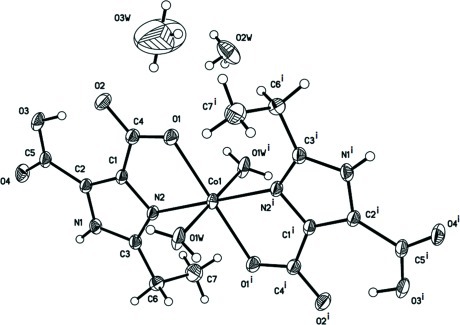
The structure of the title compound, showing the atomic numbering scheme. Non-H atoms are shown with 30% probability displacement ellipsoids. (symmetry codes: i = 1-x, -y, 1-z).

## supplementary crystallographic information

### Comment

Crystal engineering of mental-organic complexes is a very active research field. It is well known that organic ligands play a crucial role in the design and construction of desirable frameworks. In recent years, multifunctional ligands containing N– and O-donors have attracted great attention due to the fact that they may induce diversity in the coordination modes and interesting properties. In our previous work, we have done a lot of research on the design and synthesis of new compounds built from the imidazole derivatives (Fan *et al.*, 2010; Li *et al.*, 2011; He *et al.*, 2010; Song *et al.*, 2010; Yan *et al.*, 2010). To continue our study, we report here the structure of the title Co(II) complex.

As illustrated in Fig. 1, The Co^II^ ion adopts a slightly distorted octahedral geometry, with two N,O-bidentate ligands ([Co—O = 2.155 (3) Å and Co—N = 2.128 (2) Å) from the imidazoledicarboxylic group at the equatorial positions, the other two oxygen atoms (Co—O = 2.060 (2) Å) from two water molecules occupied the axial position. In the crystal structure, the complex molecules and solvent molecules are linked by O—H···O and N—H···O hydrogen bonds, forming the final three-dimensional supra-molecular network. A lattice water molecule is located on an inversion center, and one H atom of another water molecule was split into two positions with half occupancy.

### Experimental

A mixture of Co(NO_3_)_2_.6H_2_O (0.25 mmol, 0.07 g) and 2-ethyl-1*H*-imidazole-4,5-dicarboxylic acid (0.5 mmol, 0.09 g) in 10 ml of water solution was sealed in an autoclave equipped with a Teflon liner (25 ml) and then heated at 393 K for 2 d. Red crystals were obtained by slow evaporation of the solvent at room temperature with the yeild of 32% based on Co.

### Refinement

H atoms of the water molecule were located in a difference Fourier map and refined as riding with an O—H distance restraint of 0.82 (1) Å, with *U*_iso_(H) = 1.5*U*_eq_(O). The H···H distances within the water molecules were restraint to 1.30 (1) Å. Carboxyl H atoms were located in a difference map but were refined as riding on the parent O atoms with O—H = 0.82 Å and *U*_iso_(H) = 1.5 *U*_eq_(O). Carbon and nitrogen bound H atoms were placed at calculated positions and were treated as riding on the parent C or N atoms with C—H = 0.96 (methyl), 0.97 (methylene) and N—H = 0.86 Å, *U*_iso_(H) = 1.2 or 1.5*U*_eq_(C,N). The O3w is located on an inversion center, its H atoms were equally disordered over two positions. One of H atoms of O2w water molecules is also equally disordered over two sites.

### Crystal data

**Table:**

[Co(C_7_H_7_N_2_O_4_)_2_(H_2_O)_2_]·3H_2_O	*Z* = 1
*M**_r_* = 515.30	*F*(000) = 267
Triclinic, *P*1	*D*_x_ = 1.675 Mg m^−^^3^
Hall symbol: -P 1	Mo *K*α radiation, λ = 0.71073 Å
*a* = 7.1615 (14) Å	Cell parameters from 7174 reflections
*b* = 8.8729 (18) Å	θ = 2.4–28.4°
*c* = 9.3815 (19) Å	µ = 0.92 mm^−^^1^
α = 66.06 (3)°	*T* = 293 K
β = 88.66 (3)°	Block, red
γ = 70.97 (3)°	0.20 × 0.18 × 0.15 mm
*V* = 511.0 (3) Å^3^	

### Data collection

**Table:**

Bruker SMART APEXII diffractometer	2319 independent reflections
Radiation source: fine-focus sealed tube	1578 reflections with *I* > 2σ(*I*)
Graphite monochromator	*R*_int_ = 0.029
ω scans	θ_max_ = 27.5°, θ_min_ = 3.0°
Absorption correction: multi-scan (*SADABS*; Bruker, 2001)	*h* = −8→9
*T*_min_ = 0.781, *T*_max_ = 0.781	*k* = −11→11
5086 measured reflections	*l* = −12→12

### Refinement

**Table:**

Refinement on *F*^2^	Primary atom site location: structure-invariant direct methods
Least-squares matrix: full	Secondary atom site location: difference Fourier map
*R*[*F*^2^ > 2σ(*F*^2^)] = 0.035	Hydrogen site location: inferred from neighbouring sites
*wR*(*F*^2^) = 0.102	H-atom parameters constrained
*S* = 1.01	*w* = 1/[σ^2^(*F*_o_^2^) + (0.020*P*)^2^ + 1.1*P*] where *P* = (*F*_o_^2^ + 2*F*_c_^2^)/3
2319 reflections	(Δ/σ)_max_ < 0.001
149 parameters	Δρ_max_ = 0.59 e Å^−^^3^
5 restraints	Δρ_min_ = −0.73 e Å^−^^3^

### Special details

**Table:**

Geometry. All e.s.d.'s (except the e.s.d. in the dihedral angle between two l.s. planes) are estimated using the full covariance matrix. The cell e.s.d.'s are taken into account individually in the estimation of e.s.d.'s in distances, angles and torsion angles; correlations between e.s.d.'s in cell parameters are only used when they are defined by crystal symmetry. An approximate (isotropic) treatment of cell e.s.d.'s is used for estimating e.s.d.'s involving l.s. planes.
Refinement. Refinement of *F*^2^ against ALL reflections. The weighted *R*-factor *wR* and goodness of fit *S* are based on *F*^2^, conventional *R*-factors *R* are based on *F*, with *F* set to zero for negative *F*^2^. The threshold expression of *F*^2^ > σ(*F*^2^) is used only for calculating *R*-factors(gt) *etc*. and is not relevant to the choice of reflections for refinement. *R*-factors based on *F*^2^ are statistically about twice as large as those based on *F*, and *R*- factors based on ALL data will be even larger.

### Fractional atomic coordinates and isotropic or equivalent isotropic displacement parameters (Å^2^)

**Table:**

	*x*	*y*	*z*	*U*_iso_*/*U*_eq_	Occ. (<1)
Co1	0.5000	0.0000	0.5000	0.02833 (18)	
N1	0.2254 (4)	0.2967 (3)	0.7643 (3)	0.0306 (6)	
H1	0.1776	0.3183	0.8417	0.037*	
N2	0.3637 (4)	0.1497 (3)	0.6265 (3)	0.0265 (5)	
O1	0.4459 (3)	0.2642 (3)	0.3267 (3)	0.0350 (5)	
O2	0.3296 (4)	0.5449 (3)	0.2860 (3)	0.0446 (6)	
O3	0.1803 (4)	0.7116 (3)	0.4415 (3)	0.0431 (6)	
H3	0.2224	0.6487	0.3911	0.065*	
O4	0.0657 (4)	0.6633 (3)	0.6723 (3)	0.0439 (6)	
O1W	0.7752 (3)	−0.0183 (3)	0.5820 (3)	0.0461 (7)	
H1W	0.8718	−0.1140	0.6044	0.069*	
H2W	0.8118	0.0689	0.5646	0.069*	
C1	0.3196 (4)	0.3266 (4)	0.5348 (4)	0.0264 (6)	
C2	0.2335 (4)	0.4198 (4)	0.6198 (4)	0.0276 (6)	
C3	0.3052 (4)	0.1352 (4)	0.7653 (4)	0.0287 (7)	
C4	0.3683 (5)	0.3806 (4)	0.3732 (4)	0.0304 (7)	
C5	0.1529 (5)	0.6106 (4)	0.5789 (4)	0.0322 (7)	
C6	0.3153 (5)	−0.0299 (4)	0.9003 (4)	0.0359 (7)	
H6A	0.2973	−0.0090	0.9943	0.043*	
H6B	0.4462	−0.1174	0.9166	0.043*	
C7	0.1579 (7)	−0.1010 (6)	0.8754 (5)	0.0565 (11)	
H7A	0.0285	−0.0125	0.8537	0.085*	
H7B	0.1633	−0.2030	0.9686	0.085*	
H7C	0.1827	−0.1323	0.7883	0.085*	
O2W	0.1678 (5)	0.3395 (5)	0.0422 (4)	0.0726 (10)	
H3W	0.2620	0.3480	0.0885	0.109*	
H4W	0.0694	0.4355	0.0084	0.109*	0.50
H7W	0.0730	0.3366	0.0983	0.109*	0.50
O3W	0.5000	0.5000	0.0000	0.282 (8)	
H5W	0.5935	0.4424	−0.0352	0.423*	0.50
H6W	0.5155	0.4487	0.0998	0.423*	0.50

### Atomic displacement parameters (Å^2^)

**Table:**

	*U*^11^	*U*^22^	*U*^33^	*U*^12^	*U*^13^	*U*^23^
Co1	0.0327 (3)	0.0212 (3)	0.0335 (4)	−0.0056 (2)	0.0051 (3)	−0.0167 (3)
N1	0.0328 (14)	0.0297 (14)	0.0347 (15)	−0.0067 (11)	0.0064 (12)	−0.0222 (12)
N2	0.0278 (12)	0.0202 (12)	0.0323 (14)	−0.0048 (10)	0.0029 (11)	−0.0143 (11)
O1	0.0459 (13)	0.0260 (12)	0.0322 (12)	−0.0083 (10)	0.0097 (11)	−0.0149 (10)
O2	0.0673 (17)	0.0246 (12)	0.0383 (14)	−0.0143 (12)	0.0134 (13)	−0.0113 (11)
O3	0.0565 (15)	0.0228 (12)	0.0521 (16)	−0.0100 (11)	0.0074 (13)	−0.0207 (12)
O4	0.0479 (14)	0.0309 (13)	0.0554 (16)	−0.0031 (11)	0.0060 (12)	−0.0289 (12)
O1W	0.0354 (13)	0.0282 (13)	0.081 (2)	−0.0065 (10)	−0.0024 (13)	−0.0316 (13)
C1	0.0268 (14)	0.0211 (14)	0.0332 (16)	−0.0069 (12)	0.0025 (13)	−0.0144 (13)
C2	0.0261 (14)	0.0222 (15)	0.0346 (17)	−0.0057 (12)	0.0007 (13)	−0.0141 (13)
C3	0.0279 (15)	0.0267 (16)	0.0335 (17)	−0.0065 (12)	0.0017 (14)	−0.0168 (14)
C4	0.0305 (16)	0.0252 (16)	0.0348 (17)	−0.0075 (13)	0.0022 (14)	−0.0136 (14)
C5	0.0310 (16)	0.0248 (17)	0.0439 (19)	−0.0053 (13)	−0.0010 (15)	−0.0207 (16)
C6	0.0412 (18)	0.0315 (17)	0.0328 (17)	−0.0113 (15)	0.0043 (15)	−0.0125 (15)
C7	0.071 (3)	0.052 (3)	0.051 (2)	−0.034 (2)	0.006 (2)	−0.017 (2)
O2W	0.0621 (19)	0.105 (3)	0.060 (2)	−0.0130 (18)	0.0094 (16)	−0.057 (2)
O3W	0.408 (19)	0.198 (11)	0.139 (9)	−0.010 (11)	0.100 (11)	−0.052 (8)

### Geometric parameters (Å, º)

**Table:**

Co1—O1	2.153 (2)	O1W—H2W	0.8499
Co1—O1^i^	2.153 (2)	C1—C2	1.371 (4)
Co1—O1W^i^	2.064 (2)	C1—C4	1.464 (4)
Co1—O1W	2.064 (2)	C2—C5	1.481 (4)
Co1—N2	2.123 (2)	C3—C6	1.478 (4)
Co1—N2^i^	2.123 (2)	C6—C7	1.523 (5)
N1—C3	1.355 (4)	C6—H6A	0.9700
N1—C2	1.367 (4)	C6—H6B	0.9700
N1—H1	0.8600	C7—H7A	0.9600
N2—C3	1.327 (4)	C7—H7B	0.9600
N2—C1	1.377 (4)	C7—H7C	0.9600
O1—C4	1.244 (3)	O2W—H3W	0.8500
O2—C4	1.284 (4)	O2W—H4W	0.8500
O3—C5	1.292 (4)	O2W—H7W	0.8500
O3—H3	0.8500	O3W—H5W	0.8500
O4—C5	1.222 (4)	O3W—H6W	0.8500
O1W—H1W	0.8500		
			
O1W^i^—Co1—O1W	180.0	N1—C2—C1	105.4 (3)
O1W^i^—Co1—N2	90.68 (9)	N1—C2—C5	122.2 (3)
O1W—Co1—N2	89.32 (9)	C1—C2—C5	132.4 (3)
O1W^i^—Co1—N2^i^	89.32 (9)	N2—C3—N1	109.9 (3)
O1W—Co1—N2^i^	90.68 (9)	N2—C3—C6	126.0 (3)
N2—Co1—N2^i^	180.00 (9)	N1—C3—C6	124.1 (3)
O1W^i^—Co1—O1	88.57 (10)	O1—C4—O2	122.9 (3)
O1W—Co1—O1	91.43 (10)	O1—C4—C1	118.1 (3)
N2—Co1—O1	78.28 (9)	O2—C4—C1	119.0 (3)
N2^i^—Co1—O1	101.72 (9)	O4—C5—O3	124.3 (3)
O1W^i^—Co1—O1^i^	91.43 (10)	O4—C5—C2	120.1 (3)
O1W—Co1—O1^i^	88.57 (10)	O3—C5—C2	115.7 (3)
N2—Co1—O1^i^	101.72 (9)	C3—C6—C7	112.2 (3)
N2^i^—Co1—O1^i^	78.28 (9)	C3—C6—H6A	109.2
O1—Co1—O1^i^	180.0	C7—C6—H6A	109.2
C3—N1—C2	108.7 (3)	C3—C6—H6B	109.2
C3—N1—H1	125.6	C7—C6—H6B	109.2
C2—N1—H1	125.6	H6A—C6—H6B	107.9
C3—N2—C1	106.5 (2)	C6—C7—H7A	109.5
C3—N2—Co1	142.7 (2)	C6—C7—H7B	109.5
C1—N2—Co1	110.81 (19)	H7A—C7—H7B	109.5
C4—O1—Co1	114.8 (2)	C6—C7—H7C	109.5
C5—O3—H3	107.5	H7A—C7—H7C	109.5
Co1—O1W—H1W	118.0	H7B—C7—H7C	109.5
Co1—O1W—H2W	124.7	H3W—O2W—H4W	110.3
H1W—O1W—H2W	113.4	H3W—O2W—H7W	109.4
C2—C1—N2	109.5 (3)	H4W—O2W—H7W	66.5
C2—C1—C4	132.4 (3)	H5W—O3W—H6W	109.4
N2—C1—C4	118.0 (2)		
			
O1W^i^—Co1—N2—C3	91.3 (4)	C4—C1—C2—N1	179.5 (3)
O1W—Co1—N2—C3	−88.7 (4)	N2—C1—C2—C5	−178.0 (3)
N2^i^—Co1—N2—C3	−95 (100)	C4—C1—C2—C5	1.8 (6)
O1—Co1—N2—C3	179.7 (4)	C1—N2—C3—N1	0.1 (3)
O1^i^—Co1—N2—C3	−0.3 (4)	Co1—N2—C3—N1	179.4 (2)
O1W^i^—Co1—N2—C1	−89.5 (2)	C1—N2—C3—C6	177.5 (3)
O1W—Co1—N2—C1	90.5 (2)	Co1—N2—C3—C6	−3.2 (6)
N2^i^—Co1—N2—C1	84 (100)	C2—N1—C3—N2	−0.3 (3)
O1—Co1—N2—C1	−1.06 (19)	C2—N1—C3—C6	−177.7 (3)
O1^i^—Co1—N2—C1	178.94 (19)	Co1—O1—C4—O2	178.6 (2)
O1W^i^—Co1—O1—C4	92.4 (2)	Co1—O1—C4—C1	−1.4 (4)
O1W—Co1—O1—C4	−87.6 (2)	C2—C1—C4—O1	−179.3 (3)
N2—Co1—O1—C4	1.4 (2)	N2—C1—C4—O1	0.4 (4)
N2^i^—Co1—O1—C4	−178.6 (2)	C2—C1—C4—O2	0.7 (5)
O1^i^—Co1—O1—C4	−137 (100)	N2—C1—C4—O2	−179.5 (3)
C3—N2—C1—C2	0.1 (3)	N1—C2—C5—O4	−4.3 (5)
Co1—N2—C1—C2	−179.5 (2)	C1—C2—C5—O4	173.2 (3)
C3—N2—C1—C4	−179.7 (3)	N1—C2—C5—O3	176.3 (3)
Co1—N2—C1—C4	0.8 (3)	C1—C2—C5—O3	−6.3 (5)
C3—N1—C2—C1	0.3 (3)	N2—C3—C6—C7	−73.9 (4)
C3—N1—C2—C5	178.3 (3)	N1—C3—C6—C7	103.1 (4)
N2—C1—C2—N1	−0.2 (3)		

Symmetry code: (i) −*x*+1, −*y*, −*z*+1.

### Hydrogen-bond geometry (Å, º)

**Table:**

*D*—H···*A*	*D*—H	H···*A*	*D*···*A*	*D*—H···*A*
N1—H1···O2*W*^ii^	0.86	1.96	2.786 (4)	160
O3—H3···O2	0.85	1.63	2.471 (3)	171
O1*W*—H1*W*···O4^iii^	0.85	1.86	2.708 (3)	173
O1*W*—H2*W*···O3^iv^	0.85	1.94	2.763 (3)	161
O2*W*—H3*W*···O1	0.85	2.33	3.077 (4)	147
O2*W*—H3*W*···O3*W*	0.85	2.44	3.091 (3)	134
O2*W*—H4*W*···O2*W*^v^	0.85	2.04	2.883 (6)	172
O2*W*—H7*W*···O4^vi^	0.85	2.35	3.120 (4)	151
O3*W*—H5*W*···O2^vii^	0.85	2.37	3.040 (2)	136
O3*W*—H6*W*···O1	0.85	2.26	3.031 (2)	151
O3*W*—H6*W*···O2	0.85	2.43	3.040 (2)	129

Symmetry codes: (ii) *x*, *y*, *z*+1; (iii) *x*+1, *y*−1, *z*; (iv) −*x*+1, −*y*+1, −*z*+1; (v) −*x*, −*y*+1, −*z*; (vi) −*x*, −*y*+1, −*z*+1; (vii) −*x*+1, −*y*+1, −*z*.
